# Fiber supplements and clinically proven health benefits: How to recognize and recommend an effective fiber therapy

**DOI:** 10.1002/2327-6924.12447

**Published:** 2017-03-02

**Authors:** Kellen V. Lambeau, Johnson W. McRorie

**Affiliations:** ^1^ Employee and Community HealthMayo ClinicRochesterMinnesota; ^2^ Global Clinical SciencesProcter & GambleMasonOhio

**Keywords:** Dietary fiber, viscosity, large intestine, small intestine, therapeutics, nurse practitioner, advanced practice nurse

## Abstract

**Background:**

Only 5% of adults consume the recommended level of dietary fiber. Fiber supplements appear to be a convenient and concentrated source of fiber, but most do not provide the health benefits associated with dietary fiber.

**Purpose:**

This review will summarize the physical effects of isolated fibers in small and large intestines, which drive clinically meaningful health benefits.

**Data sources:**

A comprehensive literature review was conducted (Scopus and PubMed) without limits to year of publication (latest date included: October 31, 2016).

**Conclusions:**

The physical effects of fiber in the small intestine drive metabolic health effects (e.g., cholesterol lowering, improved glycemic control), and efficacy is a function of the viscosity of gel‐forming fibers (e.g., psyllium, β‐glucan). In the large intestine, fiber can provide a laxative effect if (a) it resists fermentation to remain intact throughout the large intestine, and (b) it increases percentage of water content to soften/bulk stool (e.g., wheat bran and psyllium).

**Implications for practice:**

It is important for nurse practitioners to understand the underlying mechanisms that drive specific fiber‐related health benefits, and which fiber supplements have rigorous clinical data to support a recommendation.

**Clinical pearl:**

For most fiber‐related beneficial effects, “Fiber needs to gel to keep your patients well.”

## Introduction

There are numerous fiber products on the market today. Some contain a natural fiber, such as inulin (i.e., chicory root), psyllium (i.e., husk of blond psyllium seed), or β‐glucan (i.e., oat or barley; McRorie & Fahey, 2015). Others contain an artificially created product, such as polydextrose (synthetic polymer of glucose and sorbitol), wheat dextrin (heat/acid treated wheat starch), or methylcellulose (semisynthetic, chemically treated wood pulp; McRorie & Fahey, 2015). The Institute of Medicine distinguishes *dietary fiber* (the nondigestible carbohydrates and lignin that are intrinsic and intact in plants) from *functional fiber* (the isolated, nondigestible carbohydrates that have been shown to have beneficial physiological effects in humans; Institute of Medicine, 2002). To be considered a *functional fiber*, the isolated nondigestible carbohydrate found in a fiber supplement must have clinical evidence of a beneficial physiologic effect. While the term “fiber supplement” implies that the product can help make up for a shortfall in dietary fiber consumption from whole foods such as fruits, vegetables, and whole grains, it is important for nurse practitioners to understand which supplements actually have clinical evidence of a beneficial physiologic effect and qualify as *functional fibers*.

### Background and significance

Most of what we believe about the health benefits of high dietary fiber consumption from fruits, vegetables, and whole grains comes from population‐based (epidemiologic) studies. These studies compare subpopulations (e.g., those with high vs. low dietary fiber consumption) and look for statistical associations with decreased or increased incidence of disease. The adequate intake guidelines for dietary fiber are based on a significant association between a high‐fiber diet and a reduced risk for cardiovascular disease (Institute of Medicine, 2002). The Institute of Medicine recommends a fiber intake of 14 g/1000 kcal consumed, which translates to about 25 g/day for women and 38 g/day for men (adults aged 21–50). Older adults tend to consume fewer calories, so the recommendation for women and men over 50 is 21 and 30 g/day, respectively. Only about 5% of the U.S. population achieves the recommended level of dietary fiber consumption (U.S. Department of Agriculture, 2016). On average, adults consume only about 15 g of fiber per day, and those on a low carbohydrate diet consume less than 10 g per day.

When considering the health benefits of dietary fiber (from whole foods), it is important to recognize that population‐based data lack the control necessary to establish causation. These studies can only establish statistical associations, so it is not possible to determine to what degree an observed physiologic effect is directly attributable to the fiber component of the diet, versus other health‐promoting components such as micronutrients, phytochemicals, or a reduction in fat/calorie intake. In contrast to whole foods, the physiologic effects of an isolated nondigestible carbohydrate (e.g., a fiber supplement) can be readily assessed for a direct effect in a placebo‐controlled clinical study. The purpose of this review is to provide nurse practitioners with an understanding of (a) the physical effects of isolated fibers in different regions of the gut that drive each specific health benefit, (b) which specific fibers possess the physical characteristics required to provide each specific health benefit, and (c) which specific fiber supplements are supported by rigorous evidence of a clinically meaningful health benefit.

## Health benefits derived from the physical effects of fiber in the small intestine

### Improving short‐term (postprandial) glycemic control

The small intestine is approximately 7 m long and the mucosa is studded with millions of villi, each of which is covered with approximately 1000 microvilli per 0.1 μm^2^ (i.e., brush border; McRorie & Fahey, 2015). With roughly the surface area of a tennis court, the small intestine is our largest surface area exposed to the outside world. Normally, nutrients are delivered to the small intestine within a low‐viscosity (thin) liquid called chyme that is mixed with digestive enzymes for nutrient degradation. The degraded nutrients are readily absorbed in the proximal small intestine. Introduction of a gel‐forming fiber (e.g., psyllium, β‐glucan) will significantly increase the viscosity of chyme in a dose‐dependent manner, making it thicker. This increase in viscosity slows the interactions of digestive enzymes with nutrients (slowing degradation) and slows the absorption of glucose and other nutrients (McRorie, 2015a). In the short term, this can lead to a reduced peak postprandial blood glucose concentration.

One way to assess the effects of an isolated fiber on peak postprandial blood glucose in a well‐controlled clinical study is to have subjects participate in an oral glucose tolerance test with and without a single dose of fiber. An example is a seminal study in which six healthy volunteers consumed a 50‐g glucose solution with and without several fibers, including guar gum (Jenkins et al., 1978). Raw guar gum is a highly viscous, gel‐forming fiber. When taking guar gum, the subjects had a significant decrease in peak postprandial blood glucose and insulin concentrations compared to taking liquid glucose solution alone. This beneficial effect was abolished, however, when the guar gum was hydrolyzed to a nonviscous form. Note that the commonly marketed version of guar gum is hydrolyzed to improve palatability, but this nonviscous version does not provide the viscosity/gel‐dependent health benefits of highly viscous raw guar gum. The study also compared the glycemic effects of several other gel‐forming fibers, and concluded that the fiber‐induced reduction in peak postprandial blood glucose was highly correlated with the viscosity of gel‐forming fibers (*r* = 0.926; *p* < .01; Jenkins et al., 1978). Nonviscous soluble fiber supplements (e.g., inulin, wheat dextrin, partially hydrolyzed guar gum) and insoluble fiber (e.g., wheat bran) do not provide this gel‐dependent beneficial effect (McRorie & McKeown, 2016). Wheat dextrin, an artificially created “fiber” made by altering the chemical bonds of wheat starch with heat or acid, actually resulted in an *increase* in peak postprandial blood glucose concentrations after each meal in pediatric patients being treated for type 1 diabetes and continuously monitored for blood glucose (Nader, Weaver, Eckert, & Ltief, 2014). The artificial process for turning wheat starch into wheat dextrin is incomplete, leaving some of the products readily degraded and absorbed as sugar, which resulted in higher peak postprandial blood glucose concentrations (Nader et al., 2014; Vermorel et al., 2004). It is important to note that a viscous, gel‐forming fiber can slow the absorption of nutrients, but does not reduce total nutrient absorption (Kawasaki et al., 2008). If nutrient absorption is delayed to the point where nutrients are delivered to the distal ileum, a feedback mechanism called the “ileal brake phenomenon” is stimulated, effectively slowing gastric emptying and small bowel transit to attenuate the loss of nutrients to the large intestine (McRorie & McKeown, 2016).

### Improving long‐term glycemic control in metabolic syndrome and type 2 diabetes

**Figure 1:**
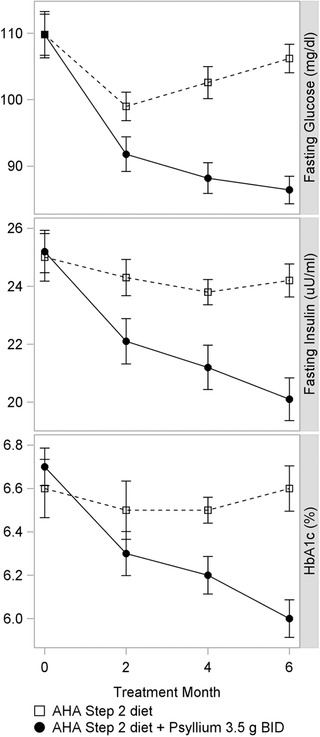
The glycemic effects over time for a 6‐month study in patients with metabolic syndrome. The controlled diet alone failed to show a sustained effect versus baseline. The addition of psyllium to the controlled diet showed improvement in glycemic measures throughout the 6‐month study.

While postprandial glucose studies are useful for assessing the acute glycemic effects of fiber, longer (multimonth) intervention studies are needed to determine if a gel‐forming fiber can provide a clinically meaningful improvement in glycemic control in patients at risk for, or being treated for, type 2 diabetes mellitus. Numerous multimonth clinical studies demonstrate a clinically meaningful reduction in fasting serum glucose, insulin, and HbA1c for a gel‐forming fiber versus placebo in patients with metabolic syndrome and type 2 diabetes (Cicero et al., 2010; Dall'Alba et al., 2013; Feinglos et al., 2013; Gibb, McRorie, Russell, Hasselblad, & D'Alessio, 2015; Tosh, 2013; Ziai et al., 2005). A 6‐month study in subjects with metabolic syndrome showed that an American Heart Association Step 2 diet was ineffective for sustained improvement glycemic control, but when psyllium (3.5 g twice a day before meals) was added to the controlled diet, fasting blood glucose, insulin, and HbA1c were all significantly reduced (Figure 1; Cicero et al., 2010). In the same study, partially hydrolyzed guar gum (same dose) showed a smaller, but still statistically significant effect. At the end of 6 months, 12.5% of the subjects in the psyllium treatment group no longer met the criteria for Metabolic Syndrome, versus only 2% in the partially hydrolyzed guar gum group, and none in the diet alone group. A placebo‐controlled study assessed the glycemic effects of psyllium (5.1 g) versus placebo (insoluble cellulose) dosed twice daily before meals for 8 weeks in patients with poorly controlled type 2 diabetes (baseline fasting blood glucose 179–208 mg/dL; baseline HbA1c 9.1–10.5%; Ziai et al., 2005). The psyllium treatment group showed significant reductions in both HbA1c (−3.0; *p* < .05) and fasting blood glucose (−89.7 mg/dL; *p* < .05) versus placebo. These gel‐dependent glycemic effects were additive to the effects already conferred by a restricted diet and stable doses of prescription drugs (a sulfonylurea and/or metformin). To optimize the glycemic effect, the gel‐forming fiber should be dosed with meals.

The effects of a gel‐forming fiber are proportional to baseline glycemic control: no effect in euglycemia (will not cause hypoglycemia); a modest effect in prediabetes (e.g. −19.8 mg/dL for psyllium 3.5 g bid; −9 mg/dL for guar gum 3.5 g bid), and the greatest effect in patients with type 2 diabetes (e.g., psyllium, −17.3 to −89.7 mg/dL; Cicero et al., 2010; Gibb et al., 2015; McRorie, 2015a; Ziai et al., 2005). A recent meta‐analysis showed that psyllium significantly improved fasting blood glucose concentration (−37 mg/dL; *p* < .001) and HbA1c (−1.0; *p* = .048) in patients being treated for type 2 diabetes (Gibb et al., 2015). Nonviscous soluble fiber (e.g., inulin, wheat dextrin), viscous nongel‐forming fiber (e.g., methylcellulose), and insoluble fiber (e.g., cellulose, wheat bran) do not provide this gel‐dependent improvement in glycemic control (McRorie & McKeown, 2016).

### Cholesterol lowering and cardiovascular health

The physical increase in chyme viscosity induced by a gel‐forming fiber can also lower elevated serum cholesterol concentrations by trapping and eliminating bile. Bile, which is released into the duodenum in response to a meal, is normally recovered in the distal ileum and recycled, potentially several times within a given meal (McRorie & Fahey, 2015). When chyme reaches the distal ileum, most of the water in the lumen has been absorbed, so a gel‐forming fiber would be more concentrated and higher in viscosity versus that in the proximal small bowel. Bile has only a short window for reuptake, so a high‐viscosity gel would significantly decrease the efficiency of reuptake, causing bile to be lost to the stool. The reduction in the bile acid pool causes hepatocytes to compensate by stimulating LDL‐receptor expression/increasing LDL‐cholesterol clearance from the blood to synthesize more bile acids (cholesterol is a component of bile) and maintain sufficient bile for digestion. This clearance of LDL cholesterol from the blood effectively lowers serum LDL cholesterol and total cholesterol (because of lowering of LDL cholesterol) concentrations, without significantly affecting HDL‐cholesterol concentration (McRorie, 2015a).

The importance of viscosity for gel‐forming fibers was demonstrated in a clinical study that assessed the cholesterol‐lowering efficacy of oat bran (β‐glucan) cereals processed to three different viscosities (high, medium, or low viscosity) in 345 subjects (LDL‐cholesterol concentrations ranged from 116 to 193 mg/dL; Wolever, Tosh, Gibbs, & Brand‐Miller, 2010). The results showed that cholesterol lowering was highly correlated with the viscosity of the gel‐forming fiber: the high‐viscosity β‐glucan (low heat and pressure processing) exhibited significant LDL cholesterol lowering (−5.5%; p<0.05 versus bran placebo), as did the medium‐viscosity (−4.7%; P<0.05), whereas the lower viscosity did not exhibit a significant cholesterol lowering effect. Another study explored the effects of processed (lower viscosity) versus nonprocessed (higher viscosity) gel‐forming oat bran on serum cholesterol in 48 subjects with hypercholesterolemia (≥200 mg/dL; Kerkhoffs, Hornstra, & Mensick, 2003). Processed oat bran (5.9 g/day β‐glucan) was baked into bread and cookies, while nonprocessed oat bran (5.0 g/day β‐glucan) was provided as raw fiber in orange juice. The processed oat bran had no significant effect on serum LDL cholesterol compared to placebo (insoluble wheat bran), while the nonprocessed oat bran, provided at a lower dose, significantly decreased LDL cholesterol (−6.7%, *p* < .001) versus placebo. Note that insoluble fiber (wheat bran) was used as a placebo. Insoluble fiber and low‐viscosity/nonviscous soluble fiber (e.g., inulin, wheat dextrin, processed β‐glucan) do not provide this viscosity/gel‐dependent beneficial effect. It should also be noted that viscosity alone, without gel‐formation, does not confer a cholesterol‐lowering benefit. A well‐controlled clinical study in 105 patients with hypercholesterolemia (total cholesterol ≥200 mg/dL) assessed the cholesterol‐lowering efficacy of a natural viscous/gel‐forming fiber (psyllium) versus a viscous but nongel‐forming semisynthetic fiber (methylcellulose; chemically altered wood pulp) and a synthetic polymer (calcium polycarbophil), all dosed three times a day before meals for 8 weeks (Anderson et al., 1991). Results showed that LDL‐cholesterol concentrations were significantly lower for the viscous/gel‐forming psyllium treatment group (−8.8%, *p* = .02 vs. placebo), but not for the methylcellulose or calcium polycarbophil treatment groups.

Psyllium has been studied in at least 24 well‐controlled clinical studies, totaling over 1500 subjects, with doses of 6–15 g/day (most studies 10 g/day; Agrawal, Tandon, & Sharma, 2007; Cicero et al., 2010; de Bock et al., 2012; Jayaram, Prasad, Sovani, Langade, & Mane, 2007; McRorie, 2015a; Moreyra et al., 2005; Ribas, Cunha, Sichieri, & da Silva, 2014; Shrestha, Freake, McGrane, Volek, & Fernandez, 2007; Vuksan et al., 2011). The studies show that psyllium lowers LDL cholesterol 6–24% and total cholesterol 2–20%, with the greatest reductions in studies with unrestricted diets and patients with high baseline cholesterol concentrations. Psyllium has also been shown to be an effective co‐therapy for statin drugs and bile acid sequestrants (Agrawal et al., 2007; Jayaram et al., 2007; McRorie, 2015a; Moreyra et al., 2005). A 3‐month study in 68 patients with hyperlipidemia showed that low‐dose simvastatin (10 mg/day) combined with psyllium (5 g tid before meals) was superior to low‐dose simvastatin alone (−63 mg/dL vs. −55 mg/dL, respectively; *p* = .03), and equivalent to a higher dose of simvastatin (20 mg/day) alone (−63 mg/dL; Moreyra et al., 2005). When combined with a bile acid sequestrant (e.g., colestipol or cholestyramine), psyllium increased the cholesterol‐lowering efficacy and decreased the symptoms associated with sequestrant therapy. These results demonstrate that a highly viscous, gel‐forming fiber supplement (e.g., psyllium) can be an effective lifestyle intervention and co‐therapy for lowering elevated serum cholesterol concentrations. Two fibers, psyllium and β‐glucan (oatmeal), have a Food and Drug Administration approved health claim for reducing the risk of cardiovascular disease by lowering serum cholesterol (Code of Federal Regulations, 2016, Title 21).

### Weight loss in patients with metabolic syndrome

In addition to improving glycemic control and lowering cholesterol, a gel‐forming fiber may also facilitate weight loss. In a 6‐month study that assessed two soluble gel‐forming fibers (guar gum and psyllium) in 141 patients with metabolic syndrome, patients were fed an American Heart Association Step 2 diet alone (control group) or the same diet supplemented with psyllium or guar gum (3.5 g twice a day before breakfast and dinner; Cicero, 2010). Both control diet and guar gum (readily fermented) showed an initial decrease in body weight, followed by weight regain over the latter months of the study. In contrast, psyllium (nonfermented) showed a sustained weight loss across the entire 6‐month test period. At the end of the 6‐month study, the psyllium, guar gum and control treatment groups lost an average of 3.3 kg, 1.6 kg and 1.2 kg versus baseline, respectively (p< .01 for psyllium versus control and guar gum). Both psyllium and guar gum showed significant improvement in fasting blood glucose (−27.9%; −11.1%), insulin (−20.4%; −10.8%), and LDL cholesterol (−7.9%; −8.5%), respectively. It is important to recognize that the fermentation process results in calorie harvest (i.e., fatty acid production/absorption), so fermentable fibers such as guar gum are not calorie‐free and may not be optimal for weight loss.

## Health benefits derived from the physical effects of fiber in the large intestine

### Improving stool form and reducing symptoms in patients with constipation, diarrhea, and irritable bowel syndrome (IBS)

It is a misconception that a high‐fiber diet will improve constipation. Not all fibers provide a laxative effect or regularity benefit, and some can even be constipating. Furthermore, it is important to recognize that the guidelines for adequate intake of fiber were based on an association between a high‐fiber diet and a reduced risk of cardiovascular disease, not a reduced risk of constipation. As concluded by the American Gastroenterological Association, “Constipation was associated with low dietary fiber intake in some, but not other studies. However, these associations do not necessarily indicate causation. Although it is reasonable to try and modify these risk factors, doing so may not improve bowel function” (Bharucha, Pemberton, & Locke, 2013, p. 219).

“Regularity” is typically defined as the regular elimination of bulky/soft/easy‐to‐pass stools (McRorie, 2015b). Constipation can be defined as infrequent (<3 bowel movements [BM] per week) elimination of small/hard stools that are difficult to pass (McRorie, 2015b). While BM frequency is often used as a measure of regularity, it should not be the primary measure. One patient may strain to pass a small, hard, “marble‐like,” stool every day (e.g., 7 BMs/week), while another may produce bulky/soft/easy‐to‐pass stools every other day (e.g., 3–4 BMs/week). In this instance, the patient with the higher BM frequency *is* constipated, while the other is not. When assessing the efficacy of increased fiber consumption, an important consideration is evidence of a significant increase in both percent stool water content (stool consistency) and stool output (assessed as grams of stool per day). The consistency of stools is dependent on stool water content, and small changes to stool water content result in large changes to stool consistency (e.g., hard stool ≤72%; normal/formed stool = 75%; soft stool 76%; loose/liquid stool ≥80% water content; McRorie, 2015b; McRorie & Fahey, 2015).

It is not feasible to separate the direct effects of fiber in a high‐fiber diet from other constituents of a high‐fiber diet (e.g., the osmotic laxative effect of sugar alcohols in fruit) on stool parameters (McRorie, 2011). In contrast, the isolated fibers found in supplements can be readily compared to a placebo in clinical studies. For an isolated fiber to exert a laxative effect/regularity benefit, it must resist fermentation to remain intact throughout the large intestine, and it must increase stool water content, which is the primary mechanism for both stool bulking and stool softening (McRorie & McKeown, 2016). Clinical studies have shown that there are two mechanisms by which an isolated fiber can exert a laxative effect: (a) insoluble fiber (e.g., poorly fermented wheat bran) remains as discreet particles (does not dissolve in water), and these discreet particles can mechanically irritate the gut mucosa, stimulating secretion of water and mucous *if* the particles are sufficiently large/coarse (fine/smooth particles can be constipating); and (b) soluble gel‐forming fiber (e.g., nonfermented psyllium) retains its high water‐holding capacity to resist dehydration throughout the large bowel (McRorie, 2015b). Both mechanisms result in bulky/soft/easy‐to‐pass stools. Psyllium has been shown to be superior to a stool softener (docusate) for increasing stool water content, stool output, and BM frequency in patients with chronic idiopathic constipation (McRorie, 2015b). Fermented fibers (e.g., inulin, polydextrose, guar gum) increase flatulence but do not provide a laxative effect/regularity benefit (McRorie & Chey, 2016). Methylcellulose (chemically altered wood pulp) has an over‐the‐counter (OTC) indication for relief of constipation, but there are no well‐controlled clinical studies in constipated patients to support efficacy versus placebo. Two fibers (soluble/fermented wheat dextrin and finely ground insoluble wheat bran) have actually been shown to *decrease* stool water content, resulting in a constipating effect (van den Heuvel et al., 2004, 2005; McRorie & Chey, 2016). Therefore, it is important for nurse practitioners to understand the mechanisms that drive a laxative effect, and to recognize which fibers have clinical evidence of a clinically meaningful laxative effect (e.g., psyllium, coarse wheat bran), versus which fibers can be constipating (e.g., wheat dextrin, finely ground wheat bran). Should a patient with chronic constipation have underlying celiac disease, it is important to note that psyllium is gluten free, and therefore provides an effective treatment option that does not risk worsening the symptoms associated with celiac disease.

In addition to effectively treating constipation, the high water‐holding capacity of nonfermented psyllium has also been shown to be effective for attenuating loose/liquid diarrheal stools (McRorie, 2015b; McRorie & McKeown, 2016; Singh, 2007) and reducing fecal incontinence episodes (Markland et al., 2015). This *stool normalizing* effect (softening hard stool in constipation and firming loose/liquid stool in diarrhea) has been shown to be effective for normalizing stool form in patients with IBS (Brandt et al., 2005; Eswaran, Muir, & Chey, 2013). For all patients, but particularly those with chronic constipation and constipation‐predominant IBS, it is important to initiate any fiber therapy gradually. As demonstrated in pain studies of IBS sufferers and healthy controls, acute distention of the bowel wall with a balloon causes sensations of bloating, discomfort and cramping pain in a step‐wise fashion. The term “cramping pain” is actually a misnomer because it is caused by passive distention of the bowel wall, not spastic contraction (McRorie, 2015b). Similar to balloon distention, introduction of fiber can generate a bolus of soft stool that, when propelled against more distal hard stool, can cause acute dilation of the bowel wall, which can be sensed as bloating/discomfort/cramping pain. To reduce the risk of fiber‐related symptoms and potentially improve long‐term compliance with an effective fiber regimen, it is important to initiate fiber therapy gradually (e.g., one dose per day for the first week, two doses per day for the second week) until the desired dose is achieved (McRorie, 2015b). Another consideration for patients with constipation is to first clear hard stool with an osmotic laxative such as magnesium citrate before initiating fiber therapy (McRorie, 2015b). Any discomfort with clearing hard stool will be associated with the osmotic laxative, potentially improving long‐term compliance with a fiber therapy regimen.

## Conclusions

Much of what we believe about the health benefits of dietary fiber is derived from population‐based epidemiologic studies, which can assess for statistical associations, but lack the control necessary to establish causation. In contrast, the isolated fibers in fiber supplements are readily assessed for a direct health effect in well‐controlled clinical studies. In the small intestine, clinical evidence supports that viscous, gel‐forming fiber (e.g., psyllium, β‐glucan) effectively lowers elevated serum cholesterol, and improves glycemic control in patients with metabolic syndrome and type 2 diabetes. Low‐viscosity/nonviscous soluble fibers (e.g., inulin, wheat dextrin) and insoluble fiber (e.g., wheat bran) do not provide these viscosity‐dependent health benefits. In the large intestine, fiber must resist fermentation to remain intact in stool and significantly increase stool water content, in order to provide a laxative effect. Large/coarse particles of insoluble wheat bran can provide a mechanically irritating effect, stimulating the mucosa to secrete water and mucous. Nonfermented gel‐forming psyllium retains its high water‐holding capacity to provide a dichotomous *stool normalizing* effect. It softens hard stool in constipation, firms loose/liquid stool in diarrhea, and normalizes stool form in patients with IBS.

## Clinical implications

While it is reasonable to recommend a high‐fiber diet, only about 5% of Americans consume the recommended level of fiber. Fiber supplements may appear to be a healthy option to increase fiber intake, but clinical evidence supports that most fibers in supplements do not provide any of the health benefits associated with a high‐fiber diet. It is therefore important for nurse practitioners to recognize the physical characteristics of isolated fibers that drive specific health benefits (e.g., viscous/gel‐forming fibers lower elevated cholesterol and improve glycemic control in type 2 diabetes). It is also important to recognize which marketed fiber supplements have rigorous clinical evidence of one or more clinically meaningful physiologic effects (Table 1). Most of the beneficial physiologic effects of fiber are gel‐dependent phenomena, and efficacy is proportional to the viscosity of the gelling fiber.

**Table 1:** Clinically meaningful effects of representative fiber supplements

	No water‐holding capacity			Water‐holding capacity			
	Insoluble	Soluble low/no viscosity		Viscous, gel‐forming			Viscous, nongelling
Fiber	Wheat bran	Wheat dextrin	Inulin	Partially hydrolyzed guar gum	β‐glucan	Psyllium	Methylcellulose
Common brand name	All‐Bran®	Benefiber®	Fiber‐Choice®	Generic	Quaker Oats®	Metamucil®	Mirafiber®, Citrucel®
Source	Wheat	Heat/acid‐treated wheat starch	Chicory root	Guar beans	Oats, barley	Seed husk, blonde psyllium	Chemically treated wood pulp
Degree of fermentation	Poorly fermented	Readily fermented	Readily fermented	Readily fermented	Readily fermented	Nonfermented	Nonfermented
Cholesterol lowering				+/−b	+	+	
Improved glycemic control				+/−b	+	+	
Constipation	+^a^					+	+/−c
Diarrhea						+	
IBS						+	

^a^If particle size is sufficiently large/coarse to stimulate the mucosa.

^b^Raw guar gum is a viscous/gel‐forming fiber, but PHGG is hydrolyzed to reduce viscosity (eliminate gelling) for improved palatability. A reduction in viscosity (loss of gel formation) correlates with a reduction in/loss of efficacy.

^c^Methylcellulose has an OTC indication for relief of constipation, but there are no well‐controlled clinical studies in constipated patients to support efficacy versus placebo. The American College of Gastroenterology determined that methylcellulose had insufficient clinical data to recommend it for treatment of chronic constipation (Brandt et al., 2005).

## References

[jaan12447-bib-0001] Agrawal, A. , Tandon, M. , & Sharma, P. (2007). Effect of combining viscous fibre with lovastatin on serum lipids in normal human subjects. International Journal of Clinical Practice, 61, 1812–1818. 17935545 10.1111/j.1742-1241.2007.01512.x

[jaan12447-bib-0038] Anderson, J. , Floore, T. , Geil, P. , Spencer, D. , & Balm, T. (1991). Hypocholesterolemic effects of different bulk‐forming hydrophilic fibers as adjuncts to dietary therapy in mild to moderate hypercholesterolemia. Archives of Internal Medicine, 151, 1597–1602. 1872664

[jaan12447-bib-0002] Bharucha, A. , Pemberton, J. , & Locke, G. (2013). American Gastroenterological Association technical review on constipation. Gastroenterology, 144(1), 218–238. 23261065 10.1053/j.gastro.2012.10.028 PMC3531555

[jaan12447-bib-0004] Brandt, L. , Prather, C. , Quigley, E. , Schiller, L. , Schoenfeld, P. , & Talley, N. (2005). Systematic review on the management of chronic constipation in North America. American Journal of Gastroenteroogy, 100, S5–S22. 10.1111/j.1572-0241.2005.50613_2.x 16008641

[jaan12447-bib-0005] Cicero, A. , Derosa, G. , Bove, M. , Imola, F. , Borghi, C. , & Gaddi, A. (2010). Psyllium improves dyslipidemaemia, hyperglycaemia and hypertension, while guar gum reduces body weight more rapidly in patients affected by metabolic syndrome following an AHA Step 2 diet. Mediterranean Journal of Nutrition and Metababolism, 3, 47–54.

[jaan12447-bib-0006] Code of Federal Regulations , Title 21. (2016). Retrieved from http://www.accessdata.fda.gov/scripts/cdrh/cfdocs/cfcfr/CFRSearch.cfm?fr=101.81

[jaan12447-bib-0007] Dall'Alba, V. , Silva, F. M. , Antonio, J. P. , Steemburgo, T. , Royer, C. , Almeida, J. , … Azevedo, M. (2013). Improvement of the metabolic syndrome profile by soluble fibre—guar gum—in patients with type 2 diabetes: A randomised clinical trial. British Journal of Nutrition, 110(9), 1601–1610. 23551992 10.1017/S0007114513001025

[jaan12447-bib-0008] de Bock, M. , Derraik, J. , Brennan, C. , Biggs, J. , Smith, G. , Cameron‐Smith, D. , … Cutfield, W. (2012). Psyllium supplementation in adolescents improves fat distribution & lipid profile: A randomized, participant‐blinded, placebo‐controlled, cross‐over trial. PLoS One, 7(7), e41735. 22848584 10.1371/journal.pone.0041735 PMC3407232

[jaan12447-bib-0009] Eswaran, S. , Muir, J. , & Chey, W. (2013). Fiber and functional gastrointestinal disorders. American Journal of Gastroenterology, 108, 718–727. 23545709 10.1038/ajg.2013.63

[jaan12447-bib-0010] Feinglos, M. , Gibb, R. , Ramsey, D. , Surwit, R. , & McRorie, J. (2013). Psyllium improves glycemic control in patients with type‐2 diabetes mellitus. Bioactive Carbohydrates and Dietary Fibre, 1, 156–161.

[jaan12447-bib-0011] Gibb, R. , McRorie, J. , Russell, D. , Hasselblad, V. , & D'Alessio, D. (2015). Psyllium fiber improves glycemic control proportional to loss of glycemic control: A meta‐analysis of data in euglycemic subjects, patients at risk of type 2 diabetes mellitus, and patients being treated for type 2 diabetes mellitus. American Journal of Clinical Nutrition, 102, 1604–1614. 26561625 10.3945/ajcn.115.106989

[jaan12447-bib-0012] Institute of Medicine, Food and Nutrition Board . (2002). Dietary reference intakes: Energy, carbohydrates, fiber, fat, fatty acids cholesterol, protein and amino acids. Washington, DC: National Academies Press.

[jaan12447-bib-0013] Jayaram, S. , Prasad, H. , Sovani, V. , Langade, D. , & Mane, P. (2007). Randomized study to compare the efficacy and safety of isapgol plus atorvastatin versus atorvastatin alone in subjects with hypercholesterolemia. Journal of the Indian Medical Association, 105(3), 142–145. 17824470

[jaan12447-bib-0014] Jenkins, D. , Wolever, T. , Leeds, A. , Gassull, M. , Haisman, P. , & Dilawari, J. (1978). Dietary fibres, fibre analogues, and glucose tolerance: Importance of viscosity. British Medical Journal, 1, 1392–1394. 647304 10.1136/bmj.1.6124.1392 PMC1604761

[jaan12447-bib-0015] Kawasaki, N. , Suzuki, Y. , Urashima, M. , Nakayoshi, T. , Tsuboi, K. , Tanishima, Y. , … & Kashiwagi, H. (2008). Effect of gelatinization on gastric emptying and absorption. Hepatogastroenterology, 55(86–87), 1843–1845. 19102405

[jaan12447-bib-0016] Kerkhoffs, D. , Hornstra, G. , & Mensick, R. (2003). Cholesterol‐lowering effect of β‐glucan from oat bran in mildly hypercholesterolemic subjects may decrease when β‐glucan is incorporated into bread and cookies. American Journal of Clinical Nutrition, 78, 221–227. 12885701 10.1093/ajcn/78.2.221

[jaan12447-bib-0017] Markland, A. , Burgio, K. , Whitehead, W. , Richter, H. , Wilcox, C. , Redden, D. , … Goode, P. (2015). Loperamide versus psyllium fiber for treatment of fecal incontinence: The fecal incontinence prescription (Rx) management (FIRM) randomized clinical trial. Diseases of the Colon and Rectum, 58, 983–993. 26347971 10.1097/DCR.0000000000000442

[jaan12447-bib-0018] McRorie, J. (2011). Prunes versus psyllium for chronic idiopathic constipation. Alimentary Pharmacology &.Therapeutics, 34, 258–259. 21679210 10.1111/j.1365-2036.2011.04713.x

[jaan12447-bib-0019] McRorie, J. (2015a). Evidence‐based approach to fiber supplements and clinically meaningful health benefits, part 2: What to look for and how to recommend an effective fiber therapy. Nutrition Today, 50(2), 90–97. 25972619 10.1097/NT.0000000000000089 PMC4415970

[jaan12447-bib-0020] McRorie, J. (2015b). Evidence‐based approach to fiber supplements and clinically meaningful health benefits, part 1: What to look for and how to recommend an effective fiber therapy. Nutrition Today, 50, 82–89. 25972618 10.1097/NT.0000000000000082 PMC4415962

[jaan12447-bib-0021] McRorie, J. , & Chey, W. (2016). Fermented fiber supplements are no better than placebo for a laxative effect. Digestive Diseases and Sciences, 61, 3140–3146. 27680987 10.1007/s10620-016-4304-1

[jaan12447-bib-0022] McRorie, J. , & Fahey, G. (2015). Fiber supplements and clinically meaningful health benefits: Identifying the physiochemical characteristics of fiber that drive specific physiologic effects In Wallace T. C. (Ed.), The CRC handbook on dietary supplements in health promotion (pp. 161–206). Florence, KY: CRC Press, Taylor & Francis Group.

[jaan12447-bib-0023] McRorie, J. , & McKeown, N. (2016). An evidence‐based approach to resolving enduring misconceptions about insoluble and soluble fiber—Understanding the physics of functional fibers in the gastrointestinal tract. Journal of the Academy of Nutrition and Dietetics. pii: S2212‐2672(16)31187‐X. doi:10.1016/j.jand.2016.09.021 [Epub ahead of print]. 10.1016/j.jand.2016.09.021 27863994

[jaan12447-bib-0024] Moreyra, A. , Wilson, A. , & Koraym, A. (2005). Effect of combining psyllium fiber with simvastatin in lowering cholesterol. Archives of Internal Medicine, 165, 1161–1166. 15911730 10.1001/archinte.165.10.1161

[jaan12447-bib-0025] Nader, N. , Weaver, A. , Eckert, S. , & Ltief, A. (2014). Effects of fiber supplementation on glycemic excursions and incidence of hypoglycemia in children with type 1 diabetes. International Journal of Pediatric Endocrinology, 13 Retrieved from http://www.ijpeonline.com/content/2014/1/13 10.1186/1687-9856-2014-13 PMC4096442 25024710

[jaan12447-bib-0026] Ribas, S. , Cunha, D. , Sichieri, R. , & da Silva, L. (2014). Effects of psyllium on LDL‐cholesterol concentrations in Brazilian children and adolescents: A randomised, placebo‐controlled, parallel clinical trial. British Journal of Nutrtition, 13, 1–8. 10.1017/S0007114514003419 25391814

[jaan12447-bib-0027] Shrestha, S. , Freake, H. , McGrane, M. , Volek, J. , & Fernandez, M. (2007). A combination of psyllium and plant sterols alters lipoprotein metabolism in hypercholesterolemic subjects by modifying the intravascular processing of lipoproteins and increasing LDL uptake. Journal of Nutrition, 137(5), 1165–1170. 17449576 10.1093/jn/137.5.1165

[jaan12447-bib-0028] Singh, B. (2007). Psyllium as a therapeutic and drug delivery agent. International Journal of Pharmacology, 334, 1–14. 10.1016/j.ijpharm.2007.01.028 17329047

[jaan12447-bib-0029] Tosh, S. M. (2013). Review of human studies investigating the postprandial blood‐glucose lowering ability of oat and barley food products. European Journal of Clinical Nutrition, 67(4), 310–317. 23422921 10.1038/ejcn.2013.25

[jaan12447-bib-0030] U.S. Department of Agriculture; Agricultural Research Service . (2016). What we eat in America: Nutrient intakes from food by gender and age (National Health and Nutrition Examination Survey (NHANES) 2009–2010). Washington, DC: Author Retrieved from http://www.ars.usda.gov/SP2UserFiles/Place/12355000/pdf/0910/Table_1_NIN_GEN_09.pdf

[jaan12447-bib-0031] van den Heuvel, E. , Wils, D. , Pasman, W. , Bakker, M. , Saniez, M. , & Kardinaal, A. (2004). Short‐term digestive tolerance of different doses of NUTRIOSE FB, a food dextrin, in adult men. European Journal of Clinical Nutrition, 58(7), 1046–1055. 15220947 10.1038/sj.ejcn.1601930

[jaan12447-bib-0032] van den Heuvel, E. , Wils, D. , Pasman, W. , Saniez, M. , & Kardinaal, A. (2005). Dietary supplementation of different doses of NUTRIOSE‐FB, a fermentable dextrin, alters the activity of faecal enzymes in healthy men. European Journal of Nutrition, 44, 445–451. 15696402 10.1007/s00394-005-0552-0

[jaan12447-bib-0033] Vermorel, M. , Coudray, C. , Wils, D. , Sinaud, S. , Tressol, J. C. , Montaurier, C. , … Rayssiguier, Y. (2004). Energy value of a low‐digestible carbohydrate, NUTRIOSE‐FB, and its impact on magnesium, calcium and zinc apparent absorption and retention in healthy young men. European Journal of Nutrition, 43, 344–352. 15309452 10.1007/s00394-004-0477-z

[jaan12447-bib-0034] Vuksan, V. , Jenkins, A. , Rogovik, A. , Fairgrieve, C. , Jovanovski, E. , & Leiter, L. (2011). Viscosity rather than quantity of dietary fibre predicts cholesterol‐lowering effect in healthy individuals. British Journal of Nutrition, 106, 1349–1352. 21736815 10.1017/S0007114511001711

[jaan12447-bib-0035] Wolever, T. , Tosh, S. , Gibbs, A. , & Brand‐Miller, J. (2010). Physicochemical properties of oat β‐glucan influence its ability to reduce serum LDL cholesterol in humans: A randomized clinical trial. American Journal of Clinical Nutrition, 92, 723–732. 20660224 10.3945/ajcn.2010.29174

[jaan12447-bib-0036] Ziai, S. , Larijani, B. , Akhoondzadeh, S. , Fakhzadeh, H. , Dastpak, A. , & Bandarian, F. (2005). Psyllium decreased serum glucose and glycosylated hemoglobin significantly in diabetic outpatients. Journal of Ethnopharmacology, 102, 202–207. 16154305 10.1016/j.jep.2005.06.042

